# Engineered basic fibroblast growth factor-overexpressing human umbilical cord-derived mesenchymal stem cells improve the proliferation and neuronal differentiation of endogenous neural stem cells and functional recovery of spinal cord injury by activating the PI3K-Akt-GSK-3β signaling pathway

**DOI:** 10.1186/s13287-021-02537-w

**Published:** 2021-08-21

**Authors:** Feifei Huang, Tianyun Gao, Wenqing Wang, Liudi Wang, Yuanyuan Xie, Chenxun Tai, Shuo Liu, Yi Cui, Bin Wang

**Affiliations:** 1 Clinical Stem Cell Center, The Affiliated Drum Tower Hospital of Nanjing University Medical School, Nanjing, 210000 China; 2 Reproductive and Genetic Center of National Research Institute for Family Planning, Beijing, 100081 China

**Keywords:** Spinal cord injury, Basic fibroblast growth factor, Mesenchymal stem cells, Gene modification

## Abstract

**Objectives:**

To investigate the safety for clinic use and therapeutic effects of basic fibroblast growth factor (bFGF)-overexpressing human umbilical cord-derived mesenchymal stem cells (HUCMSCs) in mice with completely transected spinal cord injury (SCI).

**Methods:**

Stable bFGF-overexpressing HUCMSCs clones were established by electrotransfection and then subjected to systematic safety evaluations. Then, bFGF-overexpressing and control HUCMSCs were used to treat mice with completely transected SCI by tail intravenous injection. Therapeutic outcomes were then investigated, including functional recovery of locomotion, histological structures, nerve regeneration, and recovery mechanisms.

**Results:**

Stable bFGF-overexpressing HUCMSCs met the standards and safety of MSCs for clinic use. In the mouse SCI model, stable bFGF-overexpressing HUCMSCs markedly improved therapeutic outcomes such as reducing glial scar formation, improving nerve regeneration and proliferation of endogenous neural stem cells (NSCs), and increasing locomotion functional recovery of posterior limbs compared with the control HUCMSCs group. Furthermore, bFGF-overexpressing HUCMSCs promoted the proliferation and neuronal differentiation of NSCs in vitro through the PI3K-Akt-GSK-3β pathway.

**Conclusion:**

bFGF-overexpressing HUCMSCs meet the requirements of clinical MSCs and improve evident therapeutic outcomes of mouse SCI treatment, which firmly supports the safety and efficacy of gene-modified MSCs for clinical application.

## Introduction

Spinal cord injury (SCI) is a severe, highly disabling, and fatal disease that is mostly caused by accidents [1]. SCI leads to severance of axons and death of neurons, which result in permanent functional impairments [2]. In SCI, the initial mechanical action induces the primary injury that leads to secondary injury due to numerous factors such as inflammation, ischemia, lipid peroxidation, and apoptosis [2–5]. Although the exact pathophysiological mechanisms remain unknown, preclinical studies have made major progress in neuroprotection and regeneration such as promoting long-distance axonal growth. However, no major breakthroughs translatable to therapy have been achieved and many challenges remain for basic research and clinical treatment of SCI [6, 7]. In addition to traditional management of SCI, such as medical, surgical, and rehabilitative treatments, gene therapy and cell transplantation, which are the most promising treatment options, have also been applied to SCI therapy in recent years [8, 9].

Accumulating evidence has shown that MSCs have advantages for SCI repair by reducing the inflammatory reaction, secreting nerve growth factor and neurotrophic factor, and promoting axon regeneration and nerve pathway reconstruction [10–12]. Human umbilical cord-derived mesenchymal stem cells (HUCMSCs) are easy to obtain and are widely used as seed cells in clinical trials for various diseases. Additionally, HUCMSCs have a strong proliferation ability and high transfection efficiency, which can be used as a drug vector for gene therapy [13]. MSCs therapy have emerged as a potential strategy therapy for SCI, but until today none of MSC therapies has reached its full clinical potential because the therapeutic activity of naïve seeding cells is limited, not enough to fully recover from SCI. Therefore, compared with the limited therapeutic outcomes of SCI treatment by naive HUCMSCs alone, specific gene-modified HUMSCs may be a more promising therapeutic strategy.

Basic fibroblast growth factor (bFGF; also known as FGF-2) was first extracted by Gospodarowiz from bovine brain and pituitary gland in 1974 [14]. The 18 kDa (low molecular weight) bFGF polypeptide is the active form that plays important roles in cell differentiation, proliferation, morphogenesis, angiogenesis, embryonic development, metabolism, osseous healing, and tissue repair [15]. In recent years, bFGF has been widely studied as a neuroprotective factor. A large number of studies have confirmed that bFGF plays important roles in the regeneration and repair of SCI, especially in the early stage of injury [16, 17]. Exogenous bFGF is not stable because susceptibility to heat and low pH. In vivo bFGF degrades easily and only has a 3–5-min half-life [18, 19]. In clinical trials, bFGF inactivates quickly and it is difficult to show efficacy. Thus, parenteral drug delivery is repeated to maintain an effective dose in the body. Additionally, obtaining a persistent and effective drug concentration in local lesions by systemic administration of bFGF is difficult. MSCs have the property of chemotaxis to a site of injury. To resolve the above issues, there is a need to combine gene and cell therapies to develop an effective method of promoting recovery from SCI.

For gene-modified stem cells, there is a strong concern for their safety such as tumorigenesis and tumor-enhancing effects. In previous studies, researchers have ignored systematic safety evaluation of gene-modified stem cells and did not provide evidence for safety of clinic use. In this study, we established stable bFGF-overexpressing HUCMSC clones by electrotransfection and systematically evaluated their safety and quality for clinic use. On the basis of the safety evaluation, bFGF-overexpressing HUCMSCs were used to treat mice with completely transected SCI by tail intravenous injection and the therapeutic outcomes were investigated, including locomotion recovery, histological structures, nerve regeneration, and recovery mechanisms. Our results showed that bFGF-overexpressing HUCMSCs met the standards and safety for clinic use of MSCs and markedly improved therapeutic outcomes such as reducing glial scar formation, improving nerve regeneration and proliferation of endogenous NSCs, and increasing locomotion functional recovery compared with control HUCMSC treatment. Furthermore, bFGF-overexpressing HUCMSCs promoted the proliferation and neuronal differentiation of NSCs through the PI3K-Akt-GSK-3β pathway, which potentially contributed to the beneficial effects on SCI recovery. Our study confirms the safety and efficacy of bFGF-modified HUCMSCs for clinical application.

## Materials and methods

### Plasmid construction

The PCMV3-bFGF plasmid was purchased from Sino Biological Inc. The signal peptide sequence of human interleukin-2 (IL-2) was obtained from the National Center for Biotechnology Information website. We cloned the signal peptide sequence of IL-2 to the N-terminal of human bFGF by homologous DNA technology. An automatic synthesizer was used to synthesize 60 bp of the IL-2 signal peptide sequence, and then BamHI and KpnI were used to cut the PCMV3-bFGF plasmid. The IL-2 signal sequence was ligated into PCMV3-bFGF to obtain the PCMV3-IL-2-bFGF plasmid. The nucleic acid sequence was confirmed by sequencing analysis performed at Genscript Co., Ltd.

### Isolation and culture of gene-modified HUCMSCs

This study was approved by the Research Ethics Committee of Nanjing Drum Tower Hospital. A human umbilical cord was acquired after obtaining the written informed consent by maternal donors, and HUCMSCs were isolated and cultured according to a previously published protocol [20]. Briefly, the umbilical cord tissue was cut into 2-cm segments and repeatedly washed with DPBS. Then, the umbilical cord segments were cut into small pieces (about 1 mm^3^) that were placed in a T75 culture flask. The cell culture medium (DMEM-LG + 10% qualified MSC FBS and 1% penicillin streptomycin) was added to the culture flask several hours later. Two weeks later, HUCMSC colonies appeared and fresh culture medium was replaced until the cells grew to 80–90% confluence. The HUCMSCs were digested and amplified by passaging, and the second generations of them were harvested and cryopreserved in liquid nitrogen. The samples of supernatant and cells were prepared for strict quality control and safety evaluation in accordance with our previous study [21]. After evaluation, qualified clinical HUCMSCs derived from three umbilical cords in second generation were mixed and resuscitated for following experiments to avoiding the individual heterogeneity [22]. Negative control (NC), wild-type (WT) PCMV3-bFGF, or recombinant PCMV3-IL-2-bFGF plasmids were transfected into HUCMSCs at generation 2 by electrotransfection. After transfection, the cells were seeded in 10-cm dishes under 100 µg/ml hygromycin selection. Then the screened clones that could stably express bFGF were expanded into P5-P6 generation and were cryopreserved as seeding cells. Negative control HUCMSCs were also obtained and cryopreserved at the same generation. Then, the seeding cells were resuscitated and passaged about 1–2 generations for subsequent experiments in vivo and in vitro.

### Flow cytometric analysis

To identify surface marker expression of HUCMSCs and bFGF-HUCMSCs, the cells were collected and 1 × 10^6^ cells were incubated with antibodies conjugated with fluorescein isothiocyanate (FITC) or phycoerythrin (PE), CD34-FITC, CD45-FITC, CD90-FITC, CD19-PE, CD44-PE, CD73-PE, CD105-PE, and HLA-DR-PE (BD, San Diego, CA, USA), in the dark for 15 min. After washing with PBS three times, a FACScan (BD FACSAria™; NJ, USA) and FlowJo V10 software were used to analyze the phenotype of cells.

### In vitro* differentiation assay*

HUCMSCs and bFGF-HUCMSCs were seeded in 6-well plates at a density of 1 × 10^5^ cells per well in DMEM with 10% FBS. At 80% confluence, the medium was replaced with adipogenic (Gibco, Grand Island, NY, USA) or osteogenic (Gibco) induction media to induce adipogenic and osteogenic differentiation, respectively. Differentiation induction medium was changed every 3–4 days. After 21 days of differentiation, Oil red O (Sigma-Aldrich, St. Louis, MO, USA) and Alizarin red-S (Sigma-Aldrich) staining were performed to evaluate adipogenesis and osteogenesis, respectively. Data were analyzed and quantified by ImageJ 1.52a software.

### Karyotype analysis

To validate the genetic stability of bFGF-HUCMSCs after electrotransfection, the Giemsa banding technique was used for karyotype analysis. Briefly, untransfected and stable bFGF-overexpressing HUCMSCs at sixth generation were collected and incubated at 37 °C for 30 min with 10 µg/mL colchicine solution (Roche, Basel, Switzerland). Then, the cells were fixed and spread with standard procedures. Twenty metaphases were analyzed. The number of abnormalities and morphological distortion were included as chromosomal abnormalities.

### Safety evaluations

To assess tumorigenicity, male mice with severe combined immune deficiency (SCID) were injected subcutaneously with 1 × 10^7^ bFGF-HUCMSCs at sixth generation. Human embryonic stem cells were used as a positive control [23]. We monitored and recorded the formation of tumors once a week during the observation period of 4 months. The mice were then killed and their major organs were harvested for hematoxylin–eosin (H&E) staining.

### Enzyme-linked immunosorbent assay (ELISA)

An ELISA was performed to determine bFGF levels in culture supernatants using an ELISA kit (MultiSciences Biotech Co., Ltd.) in accordance with the manufacturer’s instructions. The OD value at 450-nm wavelength was measured by an automatic enzyme immunoassay analyzer (FAME 24/20, Hamilton, Switzerland).

### RNA sequencing and transcriptome analysis

For further safety evaluation, RNA-seq technology was used to investigate whether bFGF introduction into HUCMSCs activated signaling pathways related to tumors. Total RNA was extracted from HUCMSCs and bFGF-HUCMSCs and then quantified by a NanoDrop (Thermo Fisher Scientific). Libraries were constructed and sequenced on an Illumina HiSeq X Ten platform and 150 bp paired-end reads were generated. Raw data (raw reads) in the FASTQ format were obtained by high-throughput sequencing and transformed into the original sequence by base calling. Then, quantile normalization and subsequent data processing were performed using Trimmomatic software. The transcriptome sequencing and analysis were conducted by OE biotech Co., Ltd. (Shanghai, China). DESeq software [24] was used to calculate each sample to screen the Differentially Expressed Genes ( DEGs) according to the difference multiples and the test results of the significance of the difference. *p* value < 0.05 and fold change > 2 were conditions for screening DEGs. KEGG is the main public database about pathway [25]. The KEGG pathways of DEGs were analyzed through hypergeometric test method, which could calculate result will return the *p* value of the significance of DEGs enrichment in each pathway. *p* value < 0.05 was considered statistically significant.

### SCI surgery

A total of 45 adult female C57BL/6J mice (18–20 g) were raised in a temperature and humidity-controlled environment with a light/dark cycle of 12 h/12 h for 2 weeks. After all mice were anesthetized and surgery was performed to expose the T7–9 vertebrae, the T8–9 spinal cord was completely transected with surgical blades. A total of 1 × 10^6^ HUCMSCs or bFGF-HUCMSCs were injected into a mouse through the tail vein. The animals were returned to their cage after anesthesia recovery. Experimental mice were randomly allocated to control, HUCMSC, or bFGF-HUCMSC group in accordance with the different treatments. After the operation, mice were injected with antibiotics for 3 days to prevent infections. Because of an abnormal micturition reflex, each mouse was manually micturated each day.

### Behavioral assessment

The hindlimb motor function of experimental mice was assessed by the Basso Mouse Scale (BMS) locomotor rating scale. The BMS score is a modified version of the BBB score in rats, which was designed to evaluate changes in hind limb functions of mice with spinal cord injury. The main score of the mice was used to evaluate the ankle range of motion, coordination, paw posture, trunk stability, and tail posture. At present, the BMS score has become the preferred method for functional evaluation after spinal cord injury in mice [26].

### Histological analysis

After surgery all mice were killed at 12 weeks. The spinal cords of mice were retrieved and fixed in 4% PFA. The tissues were then embedded in optimum cutting temperature compound and cut into 6–8-μm-thick sections by a Lecia CM1950 (Leica Biosystems, Wetzlar, Germany). Hematoxylin and eosin (H&E) were used to stain the tissue for general observation of cellular and extracellular matrix features. For immunofluorescence staining, the sections were incubated with primary antibodies at 4 °C overnight. The primary antibodies were glial fibrillary acidic protein (GFAP) (1:500, ab7260, Abcam), 5-hydroxytryptamine (5-HT) (1:200, 20080, Immuostar), βIII-tubulin (Tuj-1) (1:500, ab7751, Abcam, Cambridge, UK), neurofilament (NF) (1:300, ab3966, Abcam), Nestin (1:200, ab6142, Abcam), Ki-67 (1:50, ab15580, Abcam). A secondary antibody (Alexa Fluor 488, 1:500; Alexa Fluor 568, 1:500; Invitrogen, Carlsbad, CA, USA) was used to incubate sections and at last cell nuclei were stained with DAPI (ab104139, Abcam). Images were obtained under a Leica DMi8 Confocal Microscope (Leica Microsystems, Wetzlar, Germany).

### NSCs culture and treatment with conditioned media of HUCMSCs and bFGF-HUCMSCs

The hippocampus from embryos of C57BL/6 female pregnant mouse (day 12–14) was digested at 37 °C for 20 min. After that, the tissue was incubated in DMEM/F12 medium (Gibco) supplemented with 20 ng/mL epidermal growth factor, 20 ng/mL basic fibroblast growth factor (bFGF), 2% B27 (Invitrogen), and 1% penicillin–streptomycin. After two or three passages, neurospheres were digested into single cells and resuspended in DMEM/F12 medium containing 10% FBS. Then, 3.5 × 10^5^ cells were seeded on a coverslip in a 12-well plate. Moreover, 1 × 10^5^ HUCMSCs or bFGF-HUCMSCs were seeded in the upper chamber of a Transwell insert in a 12-well plate with NSCs in the lower chamber. After 24 h, the medium was replaced with differentiation medium (DMEM/F12 medium containing 2% B27) to induce the differentiation of NSCs. To observe NSC growth, the proliferation of NSCs was detected using an EDU kit (RiboBio Co., Ltd.) after coculture with HUCMSCs and bFGF- HUCMSCs in transwells. Furthermore, to detect the phosphorylation level of NSCs under stimulation of the culture supernatant of HUCMSCs, the culture supernatants of HUCMSCs and bFGF-HUCMSCs were collected using F12 medium after 48 h and then applied to serum-starved NSCs. Protein from NSCs was extracted at 15 min, 30 min, 1 h, 3 h, and 6 h for western blot analysis.

### Western blot assay

Cells were lysed with lysate buffer containing a protease inhibitor (Beyotime Biotechnology) and phosphatase inhibitor (MedChemExpress) in accordance with the manufacturers’ instructions. After extracting proteins, a BCA Protein Assay Kit (Vazyme Biotech Co., Ltd.) was used to measure the concentration. The proteins were separated by 10% sodium dodecyl sulfate polyacrylamide gel electrophoresis and then transferred to a polyvinylidene difluoride membrane (Millipore). After the membranes were blocked in 5% BSA (Sigma-Aldrich) for 2 h, primary antibodies were applied at 4 °C overnight on a horizontal shaker. The next day, the secondary antibody (1:10,000; Bioworld) was applied for 1 h at room temperature. The primary antibodies were anti-GAPDH (1:50,000; Proteintech Wuhan, China), anti-PI3K p85 (1:1000, 4257, CST), anti-PI3K p85 Y458 (1:1000, 4228, CST), anti-AKT (1:1000; ab179463, Abcam), anti-phosphorylation AKT (1:1000; ab81283, Abcam), anti-GSK3β (1:2000; ab32391, Abcam), anti-phosphorylation GSK3β S9 (1:1000; ab75814, Abcam), anti-ERK1/2 (1:2000; Proteintech, and anti-phosphorylated ERK1/2(1:1000; Beyotime). PI3K inhibitor LY294002 was purchased from MedChemExpress (HY-10108). Visualization of the protein bands was conducted with an enhanced chemiluminescence kit (Vazyme Biotech Co.,Ltd).

### Statistical analysis

Data are presented as the mean ± standard derivation (SD) of at least three replicates for each experiment. *P* < 0.05 was considered statistically significant. The data were analyzed and graphs were produced with GraphPad Prism version 5.0 software. One-way analysis of variance with Tukey’s multiple comparison test was used to analyze data.

## Results

### Construction of the bFGF plasmid and screening for stable and high expression of bFGF in HUCMSCs clones

**Fig. 1:**
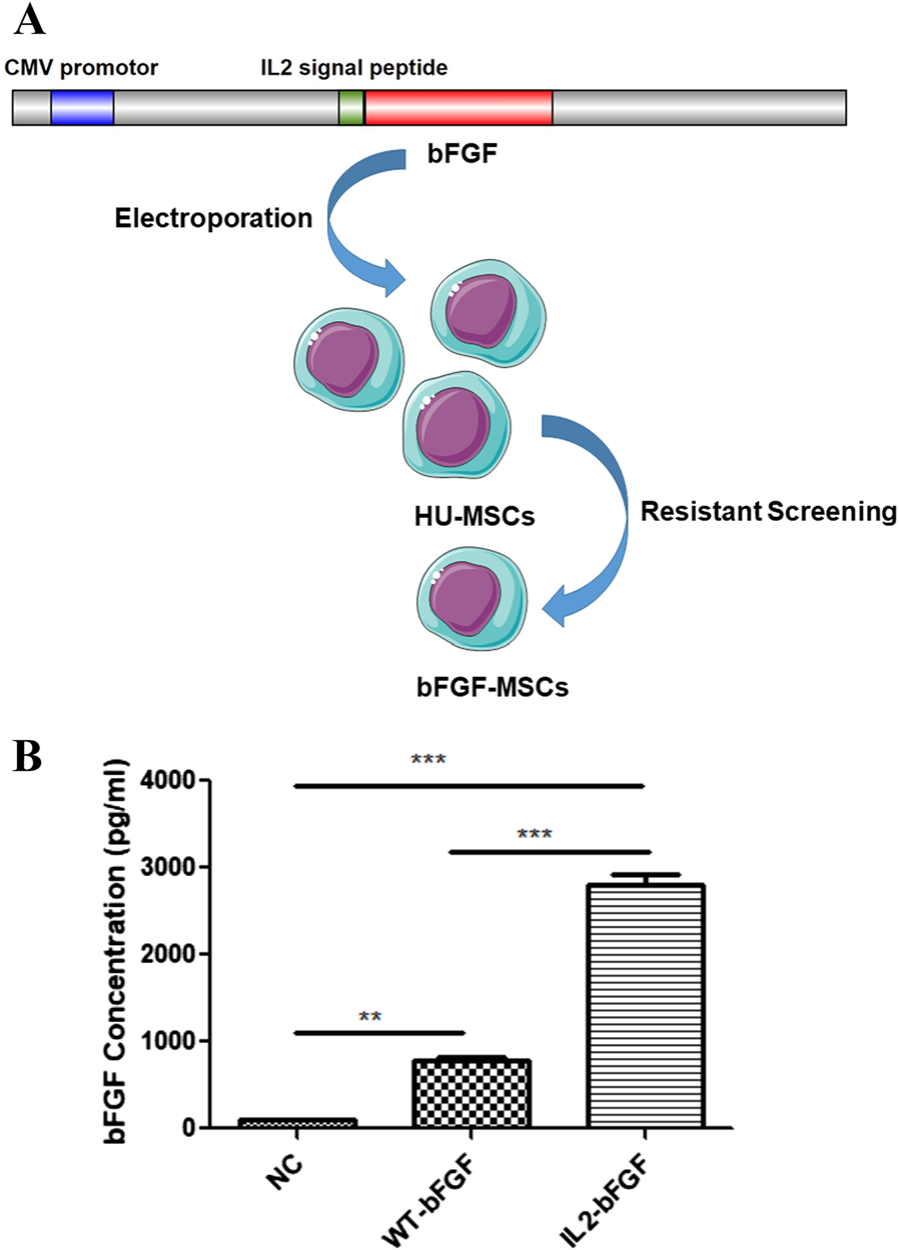
**a** Schematic diagram of plasmid construction and screening for stable bFGF-overexpressing HUCMSC clones. **b** A total of bFGF concentrations in culture supernatants of control, WT-bFGF-overexpressing, and IL-2-bFGF-overexpressing HUCMSCs. (**p < 0.01 and ***p < 0.001)

To increase bFGF secretion, a IL-2 signal peptide fragment was ligated to the N-terminal of the bFGF gene. Then, vector, wild-type bFGF, or recombinant IL-2-bFGF plasmids were transfected into HUCMSCs by nucleofection (Amaxa Kit V; Lonza). After screening for hygromycin resistance, clones were selected and expanded to stable cell lines. An ELISA was used to detect bFGF levels in culture supernatants of selected clones (Fig. 1a). In our study, 8 clones stably expressed IL2-bFGF and 5 clones stably expressed bFGF were obtained after IL2-bFGF plasmid and wild-type bFGF plasmid were electrotransfected into HUCMSCs. The IL2-bFGF and bFGF elctrotrasfection positive clones with highest expression of bFGF were used for following experiments. Control HUCMSCs only expressed a very low level of bFGF, but IL-2-bFGF-overexpressing HUCMSCs secreted a much higher level of bFGF than wild-type bFGF-overexpressing cells (Fig. 1b). In our preliminary experiment, we found that when co-cultured NSCs with IL2-bFGF-overexpressing HUCMSC and bFGF-overexpressing HUCMSC respectively, the proliferation and neuronal differentiation of NSCs were higher in IL2-bFGF-HUCMSCs than in bFGF-HUCMSCs, indicating IL2 recombinant bFGF still had the biological activity of bFGF, the better therapeutic effect of IL2-bFGF-HUCMSCs was dependent on higher secreted dose. Thus only IL-2-bFGF-overexpressing HUCMSCs were used for in cell and animal experiments. The blank vector transfection clone was used as control.

### Quality evaluation of bFGF-HUCMSCs

**Fig. 2:**
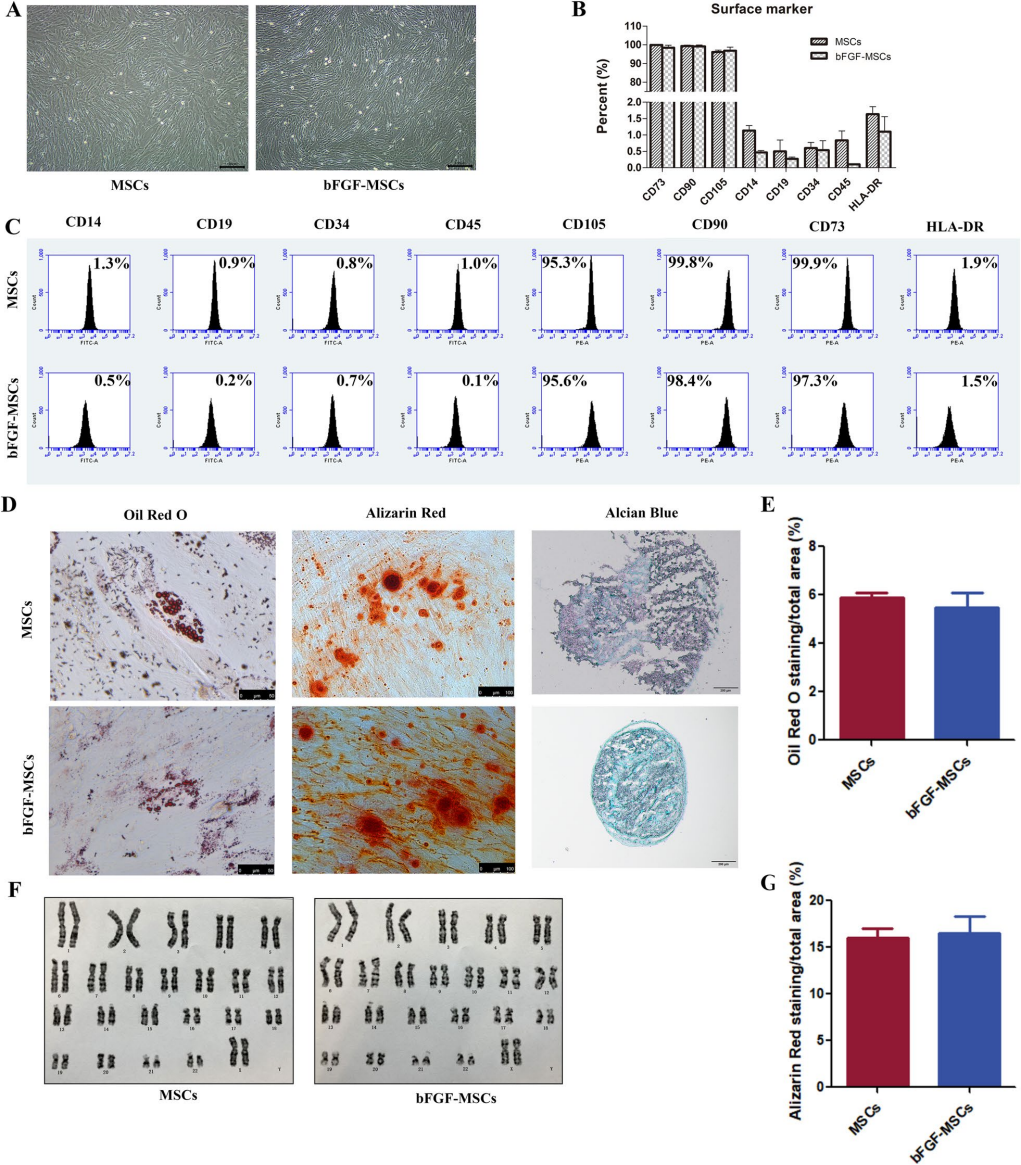
Quality evaluation of bFGF-HUCMSCs. **a** Morphology of HUCMSCs and bFGF-HUCMSCs under a light microscope. Scale bars = 100 μm. **b**, **c** Surface marker expression of MSCs and bFGF-MSCs. They both expressed CD73, CD90, and CD105, but did not express CD11b, CD19, CD34, CD45, or HLA-DR in FCM analysis. No significant differences were observed between MSCs and bFGF-HUCMSCs (*p* > 0.05). **d** Adipogenic, osteogenic, and chondrogenic differentiation was assayed by Oil Red O (Scale bars = 50 μm), Alizarin Red (Scale bars = 100 μm), and Alcian Blue (Scale bars = 200 μm) staining, respectively. **e**, **g** Quantified data of the Oil Red O staining area and Alizarin Red staining of MSCs and bFGF-MSCs by ImageJ software. **f** Karyotype analysis showed a normal karyotype (X, X) in bFGF-HUCMSCs without any karyotype variation

The quality evaluations of MSCs included morphology, surface marker expression, differentiation potential, and a normal karyotype. The morphology of stable bFGF-overexpressing HUCMSCs did not change compared with stable vector-transfected HUCMSCs (Fig. 2a). bFGF-HUCMSCs expressed markers CD105, CD90, and CD73, and did not express CD14, CD19, CD34, CD45, or HLA-DR surface markers, which was similar to HUCMSCs (Fig. 2b, c). No significant difference was observed between MSCs and bFGF-HUCMSCs (*p* > 0.05). Subsequently, osteogenic, adipogenic, and chondrogenic differentiation of bFGF-HUCMSCs was induced by respective induction medium and assayed by Alizarin Red, Oil Red O, and Alcian Blue staining, respectively. The results showed that bFGF-HUCMSCs had similar differentiation potentials to MSCs. Quantified data revealed no significant differences in the differentiation capabilities of HUCMSCs and bFGF-HUCMSCs (Fig. 2d, e, g). Transfection and overexpression of bFGF did not alter the karyotype (Fig. 2f).

### Safety of bFGF-HUCMSCs for clinical use

**Fig. 3:**
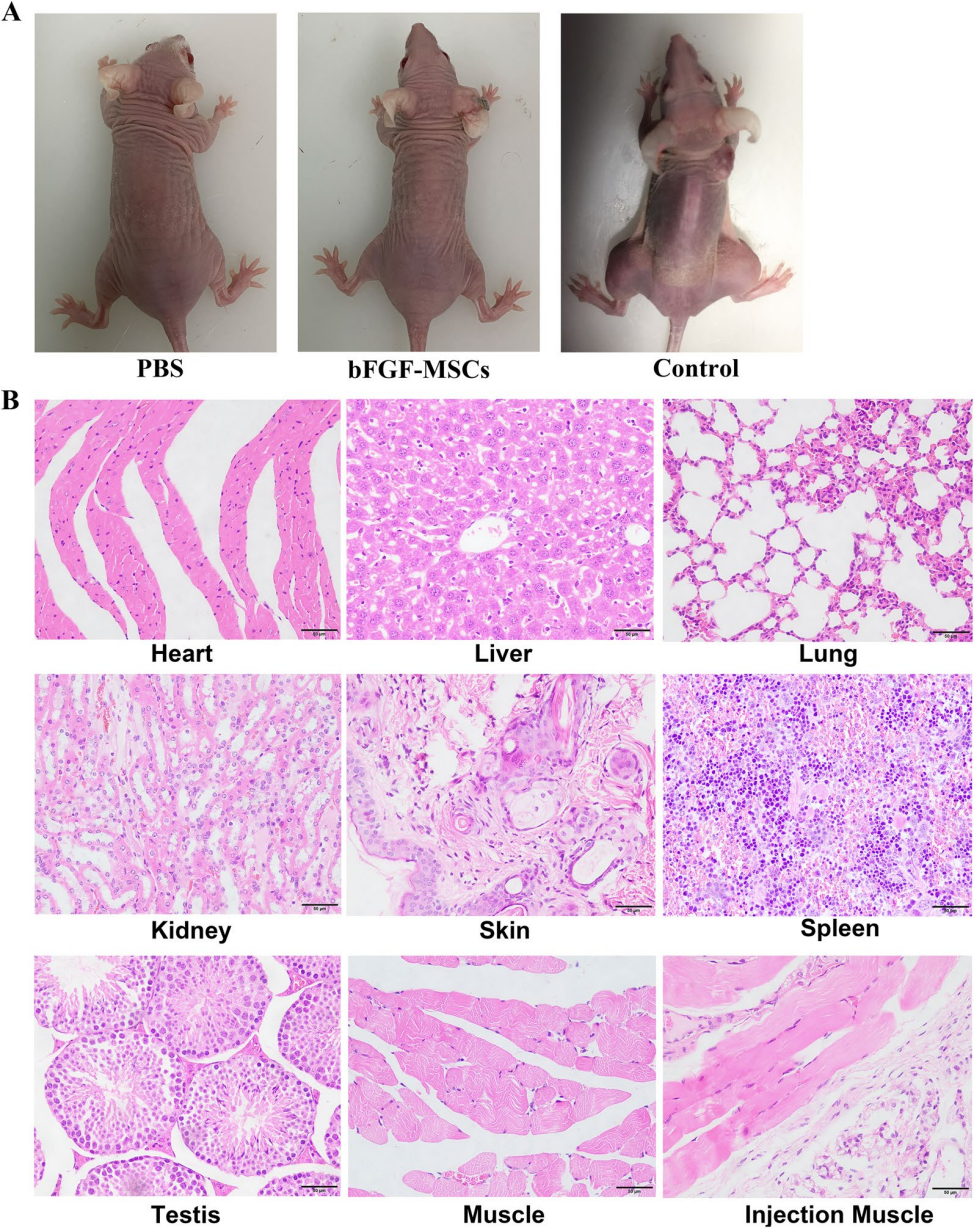
**a** Tumorigenesis of bFGF-HUCMSCs after injection into nude mice. **b** H&E staining of major organ tissues in nude mice after subcutaneous injection of bFGF-HUCMSCs. Scale bars = 50 μm

The risk of tumorigenesis is a major safety concern in stem cell-based therapy. The introduction of exogenous genes into seed cells may increase the risk of tumorigenesis to recipients. In our study, bFGF-HUCMSCs were subcutaneously injected into nude mice to test tumorigenicity and we did not subsequently observe tumorigenesis. Conversely, as a positive control, we observed obvious tumor formation at injection sites in skin after injected with human embryonic stem cells which were usually responsible for formation of tumors [23]. Staining of major organs showed that all tissues were normal without any tumor infiltration (Fig. 3a, b).

### Overexpression of bFGF in HUCMSCs does not markedly alter tumor-associated signaling pathways

**Fig. 4:**
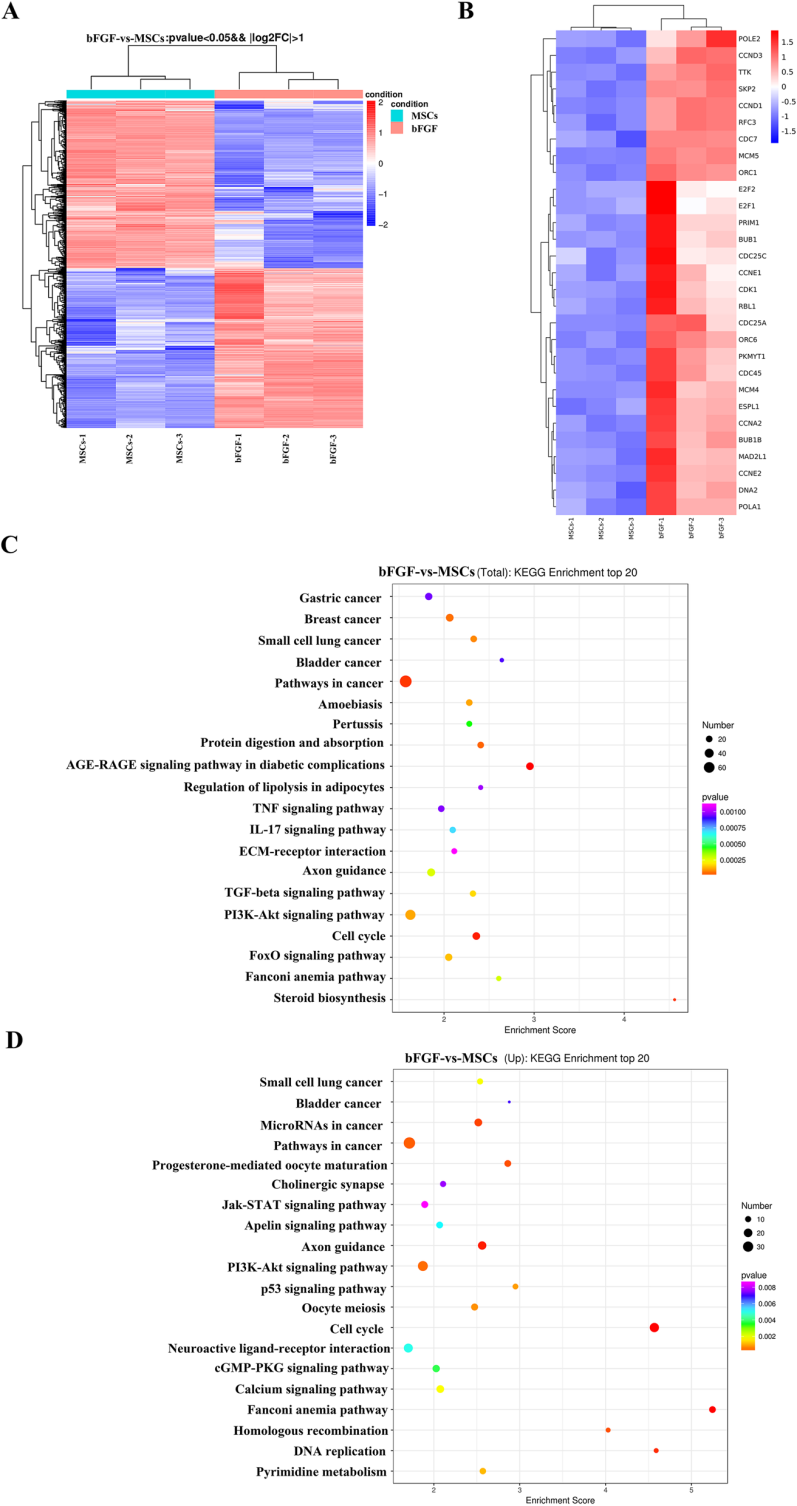
**a** Heat map of all differentially expressed genes between HUCMSCs and bFGF-HUCMSCs with a *p*-value of < 0.05. **b** Heat map of the most differentially expressed genes related to the cell cycle and DNA replication pathways between HUCMSCs and bFGF-HUCMSCs. **c** KEGG analysis of the top 20 pathways between HUCMSCs and bFGF-HUCMSCs. **d** Upregulation the 20 pathways in KEGG analysis between HUCMSCs and bFGF-HUCMSCs

Although tumorigenesis and tumor growth assays showed that our selected bFGF-MSCs clones did not induce tumor formation in nude mice, we determined whether exogenous gene insertion activated tumor-associated signaling pathways. In previous experiment, we screened out the IL2-bFGF positive clones with highest expression of bFGF by ELISA and used the clone for all experiment in the study. The data in Fig. 4a, b were from three replicates of the same clone IL2-bFGF-HUCMSCs with highest bFGF expression compared with HUCMSCs. Microarray analysis by high-throughput sequencing was used to detect differentially expressed genes between HUCMSCs and bFGF-HUCMSCs. As shown in the heat map (Fig. 4a, b), red and blue represented highly and weakly expressed genes, respectively, between HUCMSCs and bFGF-HUCMSCs. The differentially increased genes in bFGF-HUCMSCs group most were related to DNA replication and cell cycle, consistent with the biological functions of bFGF including promoting mitosis and cell proliferation(Fig. 4b) [27]. For example, POLE2 was important to DNA replication. CCND1 and CCND3 genes could promote cell proliferation. To explore the relationship between differentially expressed genes and probable pathways, KEGG pathway analysis was used to investigate the pathways enriched for the representative profiles of genes involved in signal transduction pathways (Fig. 4c). Compared with the HUCMSCs group, the 20 most remarkably changed pathways are shown in Fig. 4c. The top 20 upregulated KEGG pathways in bFGF-HUCMSCs compared with HUCMSCs were shown in Fig. 4d, of which the cell cycle and DNA replication pathways increased significant. In addition, pathway analysis also certificated that most differentially changed genes were related with PI3K-Akt signaling pathway (Fig. 4d). Although KEGG pathway analysis revealed a small increase in cancer-related pathways, the enrichment score was only slightly high basically lied in the genes related to cell proliferation, while there was no obvious upregulation of tumor biomarker genes. Therefore, bFGF gene introduction into HUC-MSCs did not remarkably activate tumor-associated genes and was safe for clinical use.

### Intravenous administration of bFGF-HUCMSCs promotes functional recovery of locomotion in the SCI model

**Fig. 5:**
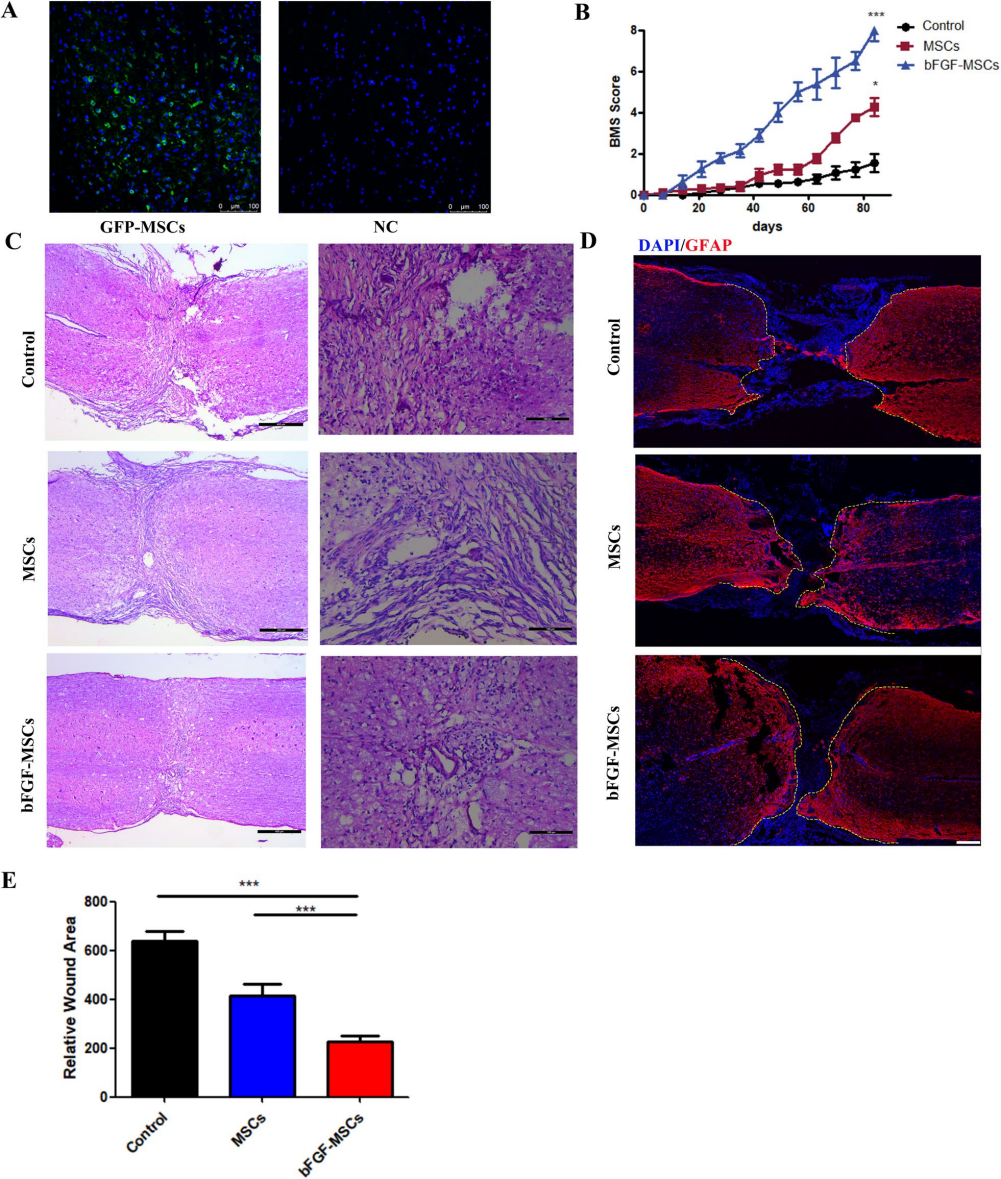
**a** HUCMSC tracing in the SCI model. Scale bars = 100 μm. **b** BMS score during the observed period post-surgery. *p < 0.05 and ***p < 0.001. **c** H&E staining of the injured site at 12 weeks post-surgery. Scale bars = 400 and 100 μm. **d**, **e** Overview of GFAP immunofluorescence staining in sections of spinal cord-transected sites. Scale bars = 200 μm. ***p < 0.001

After completely transected SCI in mice, we injected PBS, HUCMSCs, or bFGF-HUCMSCs into mice through the tail vein and then repeated the injections every 2 weeks. To determine whether HUCMSCs homed to the site of injury after administration through the caudal vein, we observed GFP-labeled HUCMSCs injected through the caudal vein after SCI. Immunostaining of GFP showed that many cells were positively stained in the injury site after GFP-labeled HUCMSC injection, which indicated that intravenously injected HUCMSCs reached the injury site for SCI repair, while no positive staining was observed after unlabeled HUCMSCs injection, which illustrated GFP-labeled HUCMSC was specific in injury site (Fig. 5a). Motor function recovery is the aim goal of SCI therapy. The Basso mouse scale (BMS) score was used to evaluate recovery of the motor functions of hind limb behavior after bFGF-HUCMSC administration in SCI mice. The mice were completely paralyzed immediately after SCI with a BMS score of 0. In the control group, spontaneous healing of hind limb paralysis was very limited and the BMS score was about 2 at 12 weeks after SCI. The BMS score in the HUCMSC treatment group increased obviously compared with the control group. However, bFGF-HUCMSC treatment remarkedly increased BMS scores at the indicated time points with significantly higher BMS scores than in control and HUCMSC groups (Fig. 5b). These results indicated that bFGF-MSC treatment led to persistent recovery of locomotor functions during the observation period. The tissue changes of the injured spinal cord at 12 weeks after SCI were observed by H&E staining. In the control group, the tissue structure of the damaged site was loose with an obvious cavity. The HUCMSC group had a larger amount of loose tissue at the injury site compared with the bFGF-HUCMSC group (Fig. 5c). Immunostaining of glial fibrillary acidic protein (GFAP) showed that GFAP-positive astrocytes were highly accumulated in rostral and caudal ends. In the control group, the area of negative astrocyte staining in the center of the damaged spinal cord was much larger compared with the HUCMSC and bFGF-HUCMSC groups (Fig. 5d). The quantified data showed that the area of the injury site was decreased sharply in the bFGF-MSC group compared with the control and MSC groups (Fig. 5e).

### Injection of bFGF-HUCMSCs enhances nerve regeneration in injured tissues after SCI

**Fig. 6:**
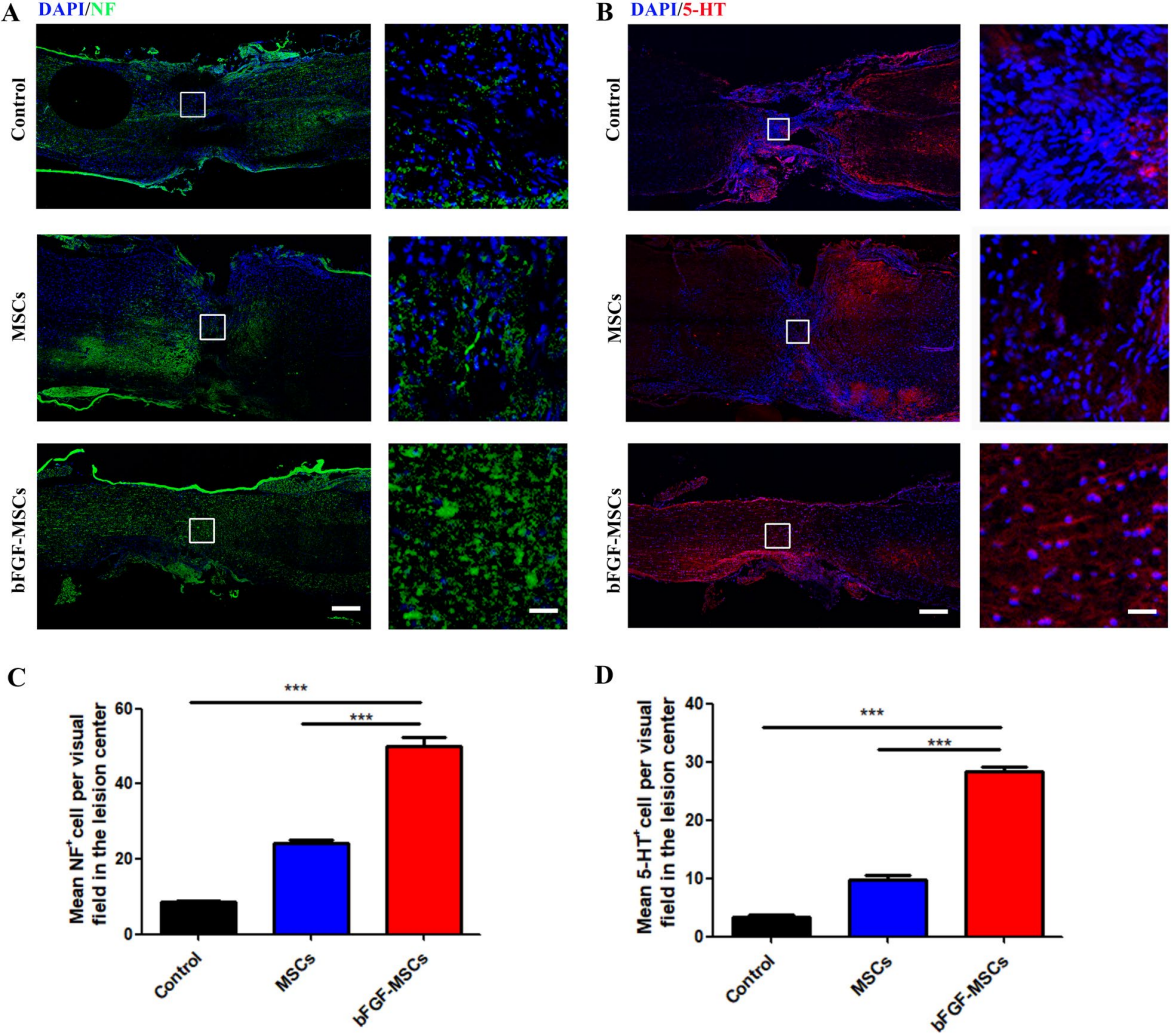
**a** Immunofluorescence staining of NF at lesion sites. Scale bars = 200 and 50 μm. **b** Immunostaining of 5-TH in injured spinal cord sections. Right panels are magnifications of boxed areas. Scale bars = 200 and 50 μm. **c** Quantification of NF staining. ***p < 0.001. **d** Quantification of 5-TH staining. ***p < 0.001

Axonal regeneration is crucial for SCI recovery. 5-Hydroxy tryptamine (5-HT) is an important neurotransmitter that participates in information transmission through the spinal cord neural network. Neurofilament (NF) is a tissue protein that exists only in neurons. Immunofluorescence staining was used to examine the regeneration of NF and 5-HT axons at 12 weeks after bFGF-HUCMSC and HUCMSCs treatments. We observed only sparse 5-HT and NF-positive cells in the center of the injured spinal cord in the control group, but obviously more positive cells in the HUCMSC treatment group. The administration of bFGF-HUCMSCs further increased 5-TH and NF-positive cells compared with the HUCMSC and control groups (Fig. 6a, b). Quantitative analysis confirmed that the number of 5-TH and NF-positive cells in the spinal cord lesion of the HUCMSC group was significantly higher than that in the control group. bFGF-HUMSC treatment remarkedly enhanced the amount of 5-TH and NF-positive cells compared with the other two groups (Fig. 6c, d).

### *BFGF-MSCs treatment remarkably accelerates the proliferation and neuronal differentiation of NSCs *in vitro* and *in vivo

**Fig. 7:**
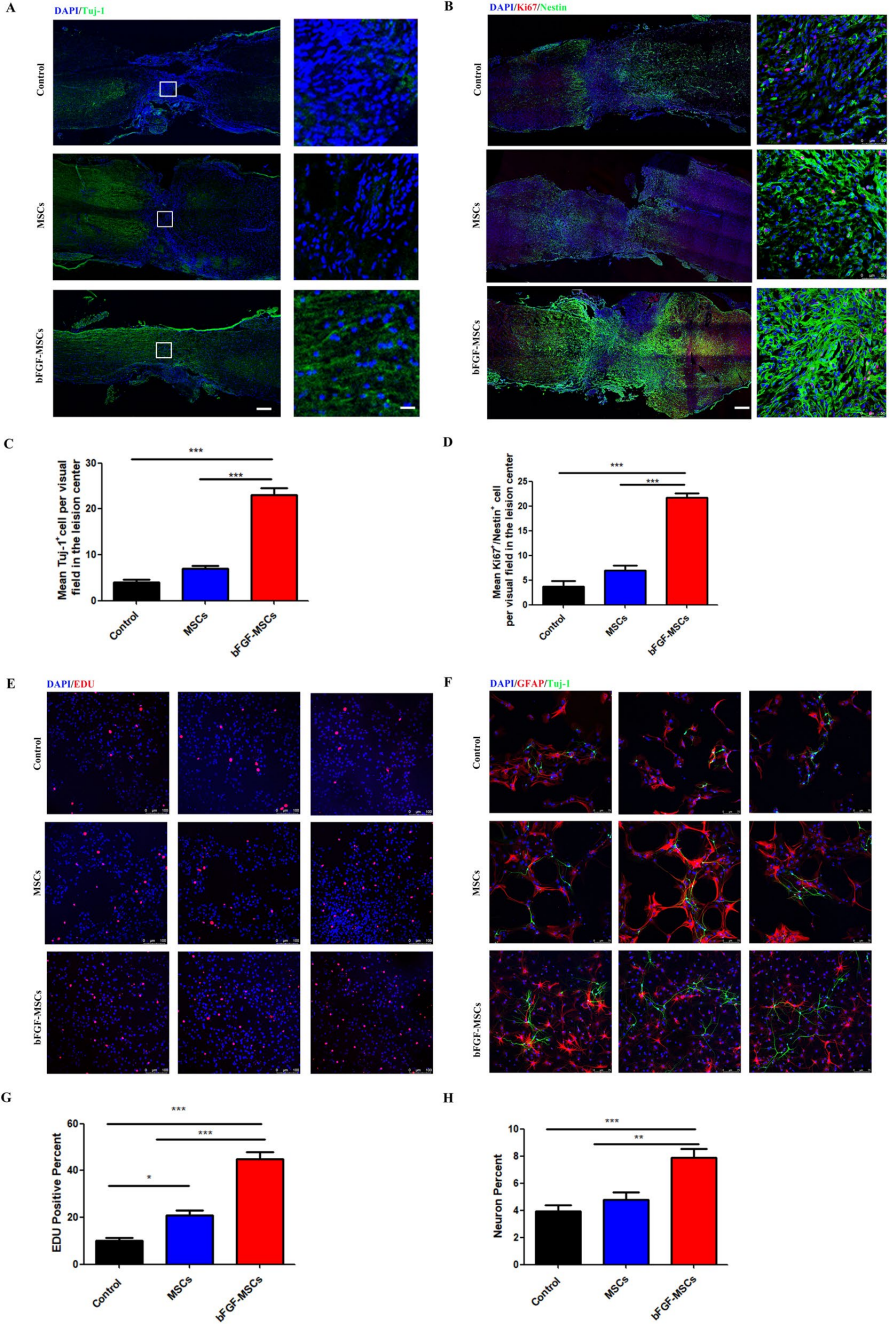
**a** Immunofluorescence staining of Tuj-1. Scale bars = 200 and 50 μm. The number of Tuj-1-positive cells was higher in the bFGF-HUCMSC group compared with the control and HUCMSC groups in the perilesional area of the injury site. **b** Immunofluorescence staining of both Ki67 and Nestin. Scale bars = 200 and 50 μm. The number of Ki67/Nestin-positive cells was higher in the bFGF-HUCMSC group compared with the control and HUCMSC groups in the perilesional area of the injury site. **c** Quantification of Tuj-1 staining in control, HUCMSC, and bFGF-HUCMSC groups. ***p < 0.001). **d** Quantification of Ki67/Nestin-positive staining. ***p < 0.001. **e** EDU staining of NSCs in vitro. Scale bars = 100 μm. **f** Immunofluorescence staining of Tuj-1 (green) and GFAP (red) in NSCs in vitro. Scale bars = 75 μm. **g** The number of EDU-positive cells in the bFGF-HUCMSC group was higher than that in the other two groups. *p < 0.05 and ***p < 0.001. **h** The number of neurons in the bFGF-HUCMSC group was higher than that in the other two groups. **p < 0.01 and ***p < 0.001

NSCs have the potential to differentiate into neurons, astrocytes, and oligodendrocytes and to self-renew sufficiently to provide cells. Tuj-1 is a neuronal marker. Immunofluorescence staining of Tuj-1 in injured spinal cord regions showed that the bFGF-HUCMSCs group had more Tuj-1-positive cells in vertical sections than control and HUCMSCs groups (Fig. 7a). Endogenous NSCs were labeled by an anti-Nestin antibody. Nestin and Ki-67 costaining represents the proliferative phase of NSCs. Double immunostaining of Nestin and Ki-67 in the injured spinal cord showed that the bFGF-MSCs group had more Nestin/Ki-67 double-positive cells in vertical sections than control and HUCMSCs groups (Fig. 7b). In vitro, EDU detection showed that the numbers of NSCs cocultured with bFGF-HUCMSCs in the transwell chamber in the proliferation period were much higher than that of NSCs cocultured with HUCMSCs under the same conditions and control NSCs (Fig. 7e, g). Immunofluorescence staining of Tuj-1 and GFAP showed that the proportion of neurons differentiated from NSCs had increased significantly when cocultured with bFGF-HUCMSCs compared with NSCs cocultured with HUCMSCs and the control group in vitro (Fig. 7f, h). Each group in Fig. 7e, f contained three duplicate samples. The multiple imagines are replica of the same experimental condition.

### Administration of bFGF-HUCMSCs activates the AKT signaling pathway in NSCs

**Fig. 8:**
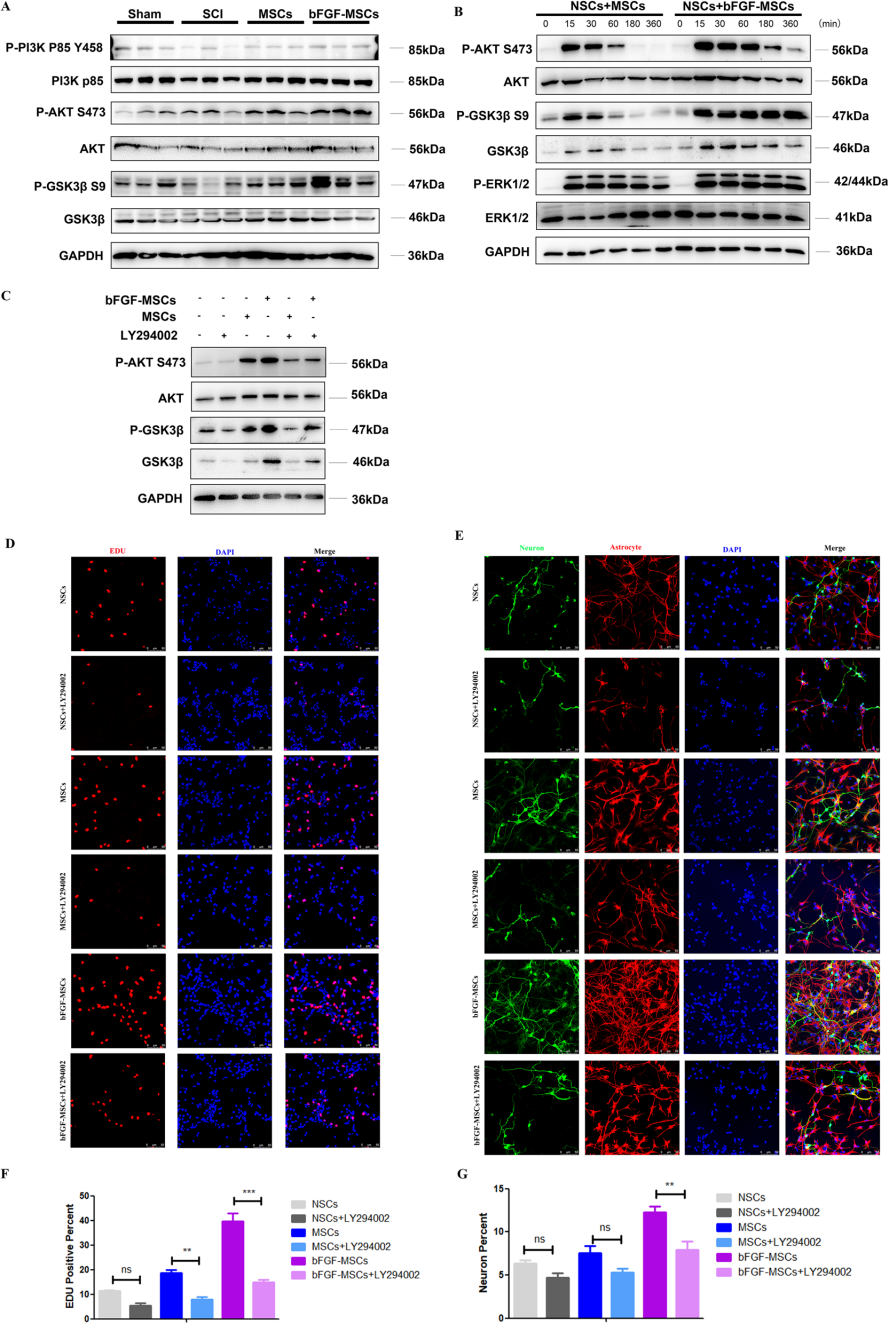
**a** Western blot analysis of the phosphorylation level of PI3K, AKT, and GSK3β in mice with an injured spinal cord. **b** Western blot analysis of the phosphorylation levels of AKT, GSK3β, and ERK1/2 in NSCs treated with HUCMSCs or bFGF-HUCMSCs culture supernatant at the indicated time points (0, 15, 30, 60, 180, and 360 min). **c** Phosphorylation levels of AKT, and GSK3β in LY294002-pretreated NSCs treated with conditioned medium of HUCMSCs or bFGF-HUCMSCs for 60 min. **d** EDU staining of LY294002-pretreated NSCs cocultured with HUMSCs or bFGF-HUCMSCs. Scale bars = 50 μm. **e** Immunofluorescence staining of Tuj-1 (green) and GFAP (red) in LY294002-pretreated NSCs cocultured with HUCMSCs or bFGF-HUCMSCs. Scale bars = 50 μm. **f**, **g** EDU staining and Tuj/GFAP staining of LY294002-pretreated NSCs cocultured with bFGF-HUCMSCs or HUCMSCs. **p < 0.01 and ***p < 0.001

We collected the injured spinal cord at 1 week after bFGF-HUCMSCs and HUMSCs treatments to investigate the mechanism of neuronal recovery. According to the results of Microarray analysis (Fig. 4d), we found that differentially changed genes between bFGF-HUCMSCs and HUCMSCs were involved in PI3K-AKT signaling pathway. For the past years, AKT signaling pathway, as a signal pathway related to various cellular functions including cell growth, reproduction, and differentiation has been studied in spinal cord injury diseases [28]. Western blot analysis showed that the phosphorylation level of PI3K, AKT, and GSK3β in the bFGF-HUCMSCs group was increased compared with the control and HUCMSCs groups, which suggested that bFGF-HUCMSCs promoted the proliferation and neuronal differentiation of NSCs by activating the PI3K-AKT-GSK3β phosphorylation pathway (Fig. 8a). Next, we collected the culture supernatants of bFGF-HUCMSCs and HUCMSCs after 48 h and then applied to serum-starved NSCs. Western blot analysis showed that the culture supernatant of bFGF-HUCMSCs increased the phosphorylation levels of AKT and GSK3β in NSCs at the indicated time points without a significant effect on the phosphorylation level of ERK1/2 (Fig. 8b). We next determined whether bFGF-HUMSCs treatment promoted the proliferation and neuronal differentiation of NSCs by activating the AKT-GSK3β signaling pathway. LY294002 is a PI3K-AKT pathway inhibitor. NSCs were pretreated with LY294002 and then treated with the culture supernatants of bFGF-HUCMSCs and HUCMSCs. The conditioned medium-induced phosphorylations of AKT and GSK3β were blocked by LY294002 treatment (Fig. 8c). Next, immunofluorescence staining was performed to examine the proliferation and neuronal differentiation of NSCs cocultured with bFGF-HUCMSCs and HUCMSCs in transwell chambers after LY294002 pretreatment. The results showed that the proliferation of NSCs cocultured with bFGF-HUCMSCs was sharply decreased by pretreatment with LY294002 (Fig. 8d, f). Similar to this phenotype, the neuronal differentiation of NSCs induced by bFGF-HUCMSCs treatment was also reduced by LY294002 pretreatment (Fig. 8e, g). Taken together, these results showed that bFGF-HUCMSCs secreted factors that promoted the proliferation and neuronal differentiation of NSCs by activating the AKT-GSK3β signal pathway.

## Discussion

SCI repair remains a great challenge worldwide and there is no effective treatment. In this study, we overexpressed bFGF in HUCMSCs for sustained high expression of bFGF at an injury site. First, bFGF-HUCMSCs met the standards and safety of MSCs for clinic use on the basis of systematic safety and quality evaluations. In the mouse SCI model, bFGF-HUCMSCs markedly improved therapeutic outcomes such as reducing glial scar formation, improving nerve regeneration and proliferation of endogenous NSCs, and increasing locomotion functional recovery of posterior limbs after repeated intravenous injections. We further found that bFGF-HUCMSCs enhanced evident therapeutic outcomes of mouse SCI treatment by promoting the proliferation and neuronal differentiation of NSCs through the PI3K-Akt-GSK-3β pathway.

The damage caused by SCI impairs the ascending and descending pathways of the spinal cord, which results in dysfunction of sensory and motor functions below the injury site [29]. MSCs have been used for SCI repair because of their various advantages. For example, MSCs release a large number of anti-inflammatory factors, cytokines, growth factors, and cell adhesion factors to change the microenvironment of SCI [30, 31]. Additionally, MSCs produce a variety of extracellular matrix proteins, promote axonal regeneration and remyelination, and induce directional growth and migration of neurons when applied to SCI [32–35]. The curative effect of MSCs is associated with paracrine activity, but has little relationship with cell replacement [36, 37]. However, for treatment of SCI, naive MSC transplantation alone is far from the ultimate goal that is complete rehabilitation after complete paralysis caused by apoptosis, inflammation, and glial scar formation after SCI. In previous literature, Tesarova et al., showed that UC-derived MSCs had higher proliferative capacity than MSCs derived from bone marrow and lipoaspirate, which was further enhanced by bFGF supplemented media [38]. Song et al. indicated that HGF which released in BMSC-CM improved the neurological function through a combination of neuron regrowth and the mediation of inflammation by blocking the BMP/Smad signaling path-way to promote neuronal differentiation of NSCs and suppressing the post-trauma inflammatory response [39]. According to these previous reports, MSCs could be combined with growth factors, other kinds of cells, drugs, and biological scaffolds to obtain satisfactory therapeutic outcomes in clinical use. Gene-modified seed cell-based therapy is becoming a promising strategy for SCI treatment by which the goal of enhancing seed cells functions or using cells to carry therapeutic factors can be achieved [8]. Additionally, MSCs have a chemotaxis feature that makes them migrate to a lesion site directly after transplantation [40]. Previous studies have shown that MSCs implanted by vein injection migrate to the center of the SCI lesion region and maintain good survival [41–43]. Thus, in our study, HUCMSCs were used as a living cell carrier to overexpress bFGF for SCI treatment. Consistent with previous studies, bFGF-HUCMSCs migrated to the lesion site, which indicated that this strategy is feasible for SCI treatment.

Studies have shown that aFGF and bFGF are widely distributed in the cytoplasm and even in the nucleus because of lacking typical secretion signal peptides [44]. bFGF plays an important regulatory role in the growth, development, differentiation, and regeneration of nerve cells [45]. Additionally, it has a vital function in the regeneration and repair of SCI, especially in the early stage. Previous studies have shown that exogenous bFGF protects the injured spinal cord and promotes regeneration of nerve fibers [46]. Furthermore, exogenous bFGF effectively inhibits the production of NOS, which reduces excessive production of NO to protect neurons. Moreover, studies have suggested that bFGF reduces the damage degree after SCI by neuroprotection, regulation of the glial response, and/or inhibiting cell apoptosis in the injured lesion to establish a beneficial microenvironment before regeneration [47–49]. However, bFGF is too large to penetrate the blood–brain barrier and its half-life is very short. Therefore, it is difficult to achieve an ideal persistent therapeutic dose of bFGF by local or systemic administration. The development of transgenic technology has enabled a new approach to treat SCI by overexpressing growth factors or neurotrophic factors in living cell carriers. In this situation, interleukin- 2 (IL2) is found as a secreted cytokine leveraging its a 20 amino acid signal peptide upstream of the mature peptide. Through studies of IL2, it is found that removing part of its N-terminal or C-terminal amino acid sequence of IL2 cDNA would lose its activity completely [50]. In this article, IL2 signal peptide sequence was introduced into the N-terminal of bFGF to obtain a new IL2-bFGF recombinant plasmid. In this way bFGF could be released outside the cell membrane and the secretion increased after gene transfection. To avoid safety concerns, we did not use virus transduction. Instead, we used electroporation to establish stable bFGF-overexpressing HUCMSCs to treat SCI, which combined the advantages of HUCMSCs and the high level of bFGF for the treatment of SCI. Our findings also confirmed that bFGF gene-modified HUMSCs remarkably improved the therapeutic outcomes of SCI treatment, which included achieving significant motor recovery, less cavity, and more Tuj-1-positive neurons and NF/5-HT-positive nerve fibers in the injured spinal cord. Axon regeneration is critical for nerve signal transmission after SCI. 5-HT nerve fibers participate in spinal cord neural network activity of vertebrate movement after SCI. These results clearly indicate that bFGF-HUCMSCs are beneficial for improving the tissue structure and regenerate neurons in the SCI area.

Gene-modified HUCMSCs must meet the standards of quality and safety when applied to clinic use. In accordance with our previous study regarding quality assays of MSCs [21], we performed systematic quality and safety evaluations of bFGF-HUCMSCs, which included morphology, surface marker expression, differential potential, karyotype, and tumorigenesis. All tests showed that bFGF-HUCMSCs met the standards and requirements of MSCs for clinic use. Previous reviews on the bFGF effect of osteogenesis and adipogenesis in MSCs had been inconsistent. It was previously observed that the extent of adipogenic and osteogenic in feline adipose MSCs staining was not influenced by the culture medium, irrespective of the bFGF addition [51]. However, some reports revealed bone marrow MSCs from elderly patients cultured with 1 ng/mL bFGF showed better differentiation potential [52]. These results may be due to the different concentration of bFGF. In our study, we found that bFGF-HUCMSCs after electroconversion could maintain the osteogenic and adipogenic capacity as MSCs. The risk of tumorigenesis is a major safety concern in stem cell-based therapy because the introduction of exogenous genes into seed cells may increase the risk of tumorigenesis in recipients. We did not observe tumorigenesis in nude mice after subcutaneous injection of bFGF-HUCMSCs. The results showed that naive HUCMSCs and bFGF-HUCMSCs did not induce the tumor formation, which indicated that the growth factors secreted from HUCMSCs and bFGF-HUCMSCs had no risk on tumor growth. Furthermore, we determined whether exogenous gene insertion activated tumor-associated signaling pathways. Microarray analysis by high-throughput sequencing showed little increase in cancer-related pathways and the enrichment score was slightly high for genes related to cell proliferation, while there were no obvious upregulation of tumor biomarker genes. Therefore, bFGF gene introduction into HUCMSCs did not remarkably activate tumor-associated genes. To our knowledge, this is first systematic quality and safety evaluation of gene-modified HUCMSCs, and bFGF-HUCMSCs had good safety for clinic use.

Additionally, we found that bFGF-HUCMSC administration promoted the proliferation and neuronal differentiation of NSCs in vivo and in vitro, which potentially contributed to the better therapeutic outcomes of SCI treatment than HUCMSC treatment. Endogenous NSCs are the source of astrocytes in glial scars, which protect tissue integrity, provide neurotrophic support for surviving neurons, and are a potential therapeutic target in SCI treatment [53]. According to the our previous experiments, Fig. 4d also showed that more genes related to PI3K-AKT signaling pathway. Previous literature also indicated bFGF could activate PI3K/Akt/GSK-3β and ERK1/2 signals to promote neuronal survival, proliferation, and anti-apoptosis [54]. The PI3K/AKT signaling pathway operates in various nerve cells, which is involved in cell growth, metabolism, and proliferation and participates in glial cell and neuronal cell survival, differentiation, apoptosis, and other processes. GSK-3β is a major downstream molecule of the PI3K/AKT pathway, and phosphorylation of GSK-3β S9 blocks GSK-3β protein activity. This signaling pathway is mainly activated by various biological factors and triggers a series of downstream signaling molecules to exert biological effects [55]. In SCI repair, activation of the PI3K/AKT signaling pathway promotes the survival of endothelial cells, reduces nerve injury and inflammatory cell death, and blocks neuron damage [56, 57]. In this study, the culture supernatant of bFGF-HUCMSCs promoted the phosphorylation of Akt and GSK-3β in NSCs in vitro, whereas increased phosphorylation levels of Akt and GSK-3β were sharply decreased after pretreatment with LY294002, a PI3K inhibitor. LY294002 was capable of suppressing PI3K/Akt/ GSK-3β pathway activity, profoundly reducing protein levels of p-Akt and p- GSK-3β. Similarly the proliferation and neuronal differentiation of NSCs cocultured with bFGF-HUCMSCs were decreased by LY294002 pretreatment in vitro. In vivo, bFGF-HUCMSCs treatment increased the PI3K, Akt, and GSK3β phosphorylation level in injured spinal cord tissue during acute injury. These results indicate that bFGF-HUCMSCs activate the PI3K/Akt signaling pathway via the secreted factors bFGF, which contributes to the proliferation and neuronal differentiation of NSCs and leads to remarkable recovery of SCI.

## Conclusions

In this study, we overexpressed bFGF in HUCMSCs and systematically evaluated their quality and safety for clinic use in accordance with the standards of clinical grade MSCs. The results showed that bFGF-HUCMSCs met the standards and safety of MSCs on the basis of strict systematic safety and quality evaluations. In the mouse SCI model, bFGF-HUCMSCs treatment markedly improved therapeutic outcomes including reducing glial scar formation, improving nerve regeneration, and increasing locomotion functional recovery of posterior limbs, which were potentially attributed to enhancing the proliferation and neuronal differentiation of NSCs by activating the PI3K-Akt-GSK-3β pathway. These results strongly indicate the feasible safety and efficacy of bFGF-modified HUMSCs for clinical application.

## Data Availability

All data generated or analyzed during this study are included in this published article. The datasets analyzed during the study are available from the corresponding author on reasonable request.

## Abbreviations

bFGF: Basic fibroblast growth factor
HUCMSCs: Human umbilical cord-derived mesenchymal stem cells
SCI: Spinal cord injury
NSCs: Neural stem cells
H&E: Hematoxylin-eosin
ELISA: Enzyme-linked immunosorbent assay
Q-PCR: Quantitative fluorescence PCR
GFAP: Glial fibrillary acidic protein
5-HT: 5-Hydroxytryptamine
Tuj-1: βIII-tubulin
NF: Neurofilament
